# Retraction Note: Antimicrobial and anti-biofilm potencies of dermcidin-derived peptide DCD-1 L against *Acinetobacter baumannii*: an in vivo wound healing model

**DOI:** 10.1186/s12866-023-03168-2

**Published:** 2024-01-04

**Authors:** Zahra Farshadzadeh, Maryam Pourhajibagher, Behrouz Taheri, Alireza Ekrami, Mohammad Hossein Modarressi, Masoud Azimzadeh, Abbas Bahador

**Affiliations:** 1 Infectious and Tropical Diseases Research Center, Health Research Institute, Ahvaz Jundishapur University of Medical Sciences, Ahvaz, Iran; 2 Department of Microbiology, School of Medicine, Ahvaz Jundishapur University of Medical Sciences, Ahvaz, Iran; 3 Dental Research Center, Dentistry Research Institute, Tehran University of Medical Sciences, Tehran, Iran; 4 Department of Medical Laboratory Sciences, School of Allied Medical Sciences, Ahvaz Jundishapur University of Medical Sciences, Ahvaz, Iran; 5 Department of Medical Genetics, School of Medicine, Tehran University of Medical Sciences, Tehran, Iran; 6 Department of Microbiology, School of Medicine, Hamadan University of Medical Sciences, Hamadan, Iran; 7 Fellowship in Clinical Laboratory Sciences, BioHealth Lab, Tehran, Iran

**Retraction note:** ***BMC Microbiol*** **22, 25 (2022)**

10.1186/s12866-022-02439-8

The Editor has retracted this article. After publication, concerns were raised regarding image similarities in the presented figures. Specifically:

- Figure 4a and b appear to overlap;
- Figure 5a and b appear to overlap (both low and high magnification images);
- Figure 7c, d and e appear to originate from the same sample.

The authors have provided partial raw data to address these concerns. Further checks by the Publisher have found that in addition to the above concerns, Fig. 4c-f images all appear to originate from the same sample.

The Editor therefore no longer has confidence in the presented data.

Abbas Bahador does not agree to this retraction. Maryam Pourhajibagher and Alireza Ekrami have not responded to any correspondence from the publisher about this retraction. The publisher has not been able to obtain current email addresses for Zahra Farshadzadeh, Behrouz Taheri, Mohammad Hossein Modarressi and Masoud Azimzadeh.

